# Genetic Drift and Purifying Selection Shaped Mitochondrial Genome Variation in the High Royal Jelly-Producing Honeybee Strain (*Apis mellifera ligustica*)

**DOI:** 10.3389/fgene.2022.835967

**Published:** 2022-02-09

**Authors:** Chuan Ma, Ruoyang Hu, Cecilia Costa, Jianke Li

**Affiliations:** ^1^ Institute of Apicultural Research, Chinese Academy of Agricultural Sciences, Beijing, China; ^2^ CREA Research Centre for Agriculture and Environment, Bologna, Italy

**Keywords:** genetic drift, mtDNA copy number, positive selection, purifying selection, royal jelly

## Abstract

Mitochondrial genomes (mitogenomes) are involved in cellular energy metabolism and have been shown to undergo adaptive evolution in organisms with increased energy-consuming activities. The genetically selected high royal jelly-producing bees (RJBs, *Apis mellifera ligustica*) in China can produce 10 times more royal jelly, a highly nutritional and functional food, relative to unselected Italian bees (ITBs). To test for potential adaptive evolution of RJB mitochondrial genes, we sequenced mitogenomes from 100 RJBs and 30 ITBs. Haplotype network and phylogenetic analysis indicate that RJBs and ITBs are not reciprocally monophyletic but mainly divided into the RJB- and ITB-dominant sublineages. The RJB-dominant sublineage proportion is 6-fold higher in RJBs (84/100) than in ITBs (4/30), which is mainly attributable to genetic drift rather than positive selection. The RJB-dominant sublineage exhibits a low genetic diversity due to purifying selection. Moreover, mitogenome abundance is not significantly different between RJBs and ITBs, thereby rejecting the association between mitogenome copy number and royal jelly-producing performance. Our findings demonstrate low genetic diversity levels of RJB mitogenomes and reveal genetic drift and purifying selection as potential forces driving RJB mitogenome evolution.

## Introduction

As the major source of cellular energy, mitochondria produce roughly 90% of the energy in the form of ATP through oxidative phosphorylation (OXPHOS) (Schon et al., 2012; Castellani et al., 2020). Mitochondria contain their own DNA (mtDNA), a maternally inherited genome (mitogenome), which in metazoans generally contains 37 genes. Of these genes, 13 encode subunits of protein complexes directly involved in OXPHOS and 24 encode genes (two ribosomal RNAs and 22 transfer RNAs) for the mitochondrial translational machinery. Hundreds to several thousands of mtDNA copies are present in a cell and the copy number is associated with OXPHOS enzyme activity and ATP production ability (Jeng et al., 2008). Accumulating evidence indicates variations in mtDNA copy number depending on physiological conditions and energy demands (Schon et al., 2012; D'Erchia et al., 2015; Castellani et al., 2020).

Mitogenome evolution could be driven by various selective forces. An important role is played by purifying selection, which can reduce genetic diversity by eliminating deleterious mutations to maintain proper protein function (Palozzi et al., 2018). Strong purifying selection acting on mitogenomes has been observed in animals with elevated energy requirements, such as rapidly flying birds (Shen et al., 2009) and fish with migratory ability (Sun et al., 2011). In smaller populations, however, purifying selection is less effective to sweep away deleterious mutations. Consequently, some slightly deleterious mutations could become more frequent and even fixed in a population due to chance, through a process known as genetic drift (Pavlova et al., 2017). Founder effects, a special form of genetic drift, occur when a new population is established by a limited number of individuals (founder population) randomly derived from a larger original population (Weaver et al., 2021). By contrast, beneficial mutations that increase survival or reproductive fitness will become selectively fixed in a population. Such positive selection has been identified in mitochondrial genes of animals with stronger energy-consuming activities, e.g., flying insects (Yang et al., 2014; Mitterboeck et al., 2017; Li et al., 2018) and bats (Shen et al., 2010). Disentangling the underlying forces shaping mitogenome variation has become a focus of recent comparative mitogenomics studies (Sun et al., 2018; Wei et al., 2020).

The high royal jelly-producing honeybee strain (from hereon “RJB”) in China has been derived from Italian bees (ITBs, *Apis mellifera ligustica*) by selective breeding for high royal jelly yield (Cao et al., 2016; Altaye et al., 2019). The annual royal jelly yield is now more than 10 kg per RJB colony, which is 10 times higher than that of ITBs (Altaye et al., 2019; Hu et al., 2019). The primary constituents of royal jelly, which include water, proteins, and small-molecule compounds, still maintain similar levels in RJBs as in ITBs (Li et al., 2007; Ma et al., 2021). To achieve the high performance of royal jelly production, adaptive changes in nuclear genome sequences (Wragg et al., 2016; Rizwan et al., 2020) and tissues/organs related to royal jelly synthesis and secretion have occurred in RJBs. Notably, RJBs possess larger and more numerous acini and a higher secretory activity of the hypopharyngeal glands (Li et al., 2010; Cao et al., 2016). Furthermore, pathways involving protein synthesis and energy metabolism are highly activated in the brain (Han et al., 2017; Zhang et al., 2020), hypopharyngeal glands (Hu et al., 2019), mandibular glands (Huo et al., 2016), hemolymph (Ararso et al., 2018), and antennae (Wu et al., 2019) of RJBs. These observations indicate a much higher energy demand in RJBs.

Considering the vital role of mitogenome in energy metabolism, it appears reasonable to hypothesize that mitogenome copy number and/or sequence variations could underlie metabolic adaptations to the improved royal jelly yield in RJBs. However, such comparative investigations are still limited (Ma and Li, 2021), e.g., based on ∼1200-bp mtDNA sequence of partial *nad2* and *cox1*−*cox2* genes (Cao et al., 2017). Here, to test for potential adaptive evolution of RJB mitogenomes, we sequenced and compared mitogenomes from 100 RJBs sampled from the main royal jelly-producing areas in China and 30 ITBs not selected for high royal jelly production across Italy. To this aim, we examined genetic diversity and phylogenetic positions of RJBs from a mitogenome viewpoint, explored selection patterns acting on mitochondrial genes, and quantified mtDNA copy abundance in the brain, hypopharyngeal glands, and mandibular glands. Our study provides insights into potential driving forces that have shaped RJB mitogenome evolution.

## Materials and Methods

### Royal Jelly Production

ITB (queens from Bologna, Italy) and RJB (queens from Zhejiang, China) colonies were raised at the apiary of the Institute of Apicultural Research, Chinese Academy of Agricultural Sciences in Beijing, China. For each strain, six colonies with similar population structure and food storage were selected for royal jelly production following traditional procedures (Altaye et al., 2019). Briefly, 126 young worker larvae (<24 h after hatching) were grafted into plastic queen cells and placed into the queenless chamber super of a colony. At 72 h post-grafting, the royal jelly in each colony was collected and weighed with a digital balance scale (0.1 mg in accuracy; Mettler-Toledo, Germany).

### mtDNA Copy Number Quantification

Bees that were displaying typical nursing behavior, i.e., head in a worker cell for at least 3 s (Jasper et al., 2015), were collected from the above colonies to quantify mtDNA copy number. Three tissues including the brain, hypopharyngeal glands, and mandibular glands, which are involved in royal jelly synthesis and secretion (Altaye et al., 2019), were dissected respectively from these nurse bees. For each tissue, 3−5 bees from a colony were pooled to form a biological sample and six biological samples were prepared for each honeybee strain. Whole genomic DNA was extracted from each sample using a DNeasy Blood & Tissue kit (Qiagen, Hilden, Germany), and potential RNA was removed using RNase A (Solarbio, Beijing, China). DNA quality and concentration were measured using a NanoDrop 2000 Spectrophotometer (Thermo scientific, DE, United States).

mtDNA copy number was determined as the relative amount of a mitochondrial gene (16S rRNA) to a single-copy nuclear gene (glyceraldehyde-3-phosphate dehydrogenase, GAPDH) using quantitative real-time (qRT)-PCR. The forward and reverse primers designed and used in our study were 5′-AGA AAC CAA TCT GAC TTA CG and 5′-ATT ACC TTA GGG ATA ACA GC for 16S rRNA gene region and 5′-CTT ACA GTT ATG GCG AGA C and 5′-ATT CCT TTC AAT GGT CCT TC for GAPDH. Each reaction was performed in technical duplicates with TB Green^®^ *Premix Ex Taq*™ II (TaKaRa, Dalian, China) and fixed amount of DNA templates (30 ng for brain, 100 ng for hypopharyngeal glands, and 10 ng for mandibular glands). The amplification was performed on the LightCycler 480 (Roche Applied Science, Penzberg, Germany) under conditions (95°C for 30 s; 40 cycles of 95°C for 5 s, 55°C for 5 s, and 72°C for 15 s). To verify the specificity of the primers, a melt curve analysis was conducted and the resultant qRT-PCR products were sequenced. Cycle threshold (Ct) values were obtained from the qRT-PCR and the relative mtDNA copy number was calculated as 2 × 2^ΔCt^, where ΔCt = Ct_GAPDH_ − Ct_16S rRNA_ (Leuthner et al., 2021).

### Mitogenome Sequencing and Annotation

A total of 100 drone pupae of RJBs were collected from 20 apiaries (five from each apiary) in Zhejiang and Jiangsu, China, whereas 30 adult drones of ITBs unselected for royal jelly production were collected from 23 apiaries across Italy (Supplementary Table S1). Individual drones were sampled from different colonies (i.e., one drone per colony). These samples were preserved in 100% ethanol at −20°C. Genomic DNA was extracted from each sample using a DNeasy Blood & Tissue kit (Qiagen, Hilden, Germany). The DNA was used for 150-bp paired-end sequencing on the Illumina HiSeq 2500 platform (Novogene, Beijing, China). Raw reads were trimmed with the Trimmomatic preprocessing tool (v0.39) (Bolger et al., 2014) and the obtained clean reads were assembled by MitoZ (v2.4-alpha) (Meng et al., 2019). The 130 mitogenomes were annotated on the MITOS webserver (Bernt et al., 2013) with the invertebrate mitochondrial code. Gene boundaries were manually refined based on homologous gene annotations for other *A*. *mellifera* mitogenomes available in GenBank.

### Genetic Diversity

Each of the 37 genes of the 130 mitogenomes was aligned using MUSCLE (Edgar, 2004) and concatenated into a single dataset for genetic diversity analysis. Genetic diversity was assessed by the number of haplotypes and polymorphic sites, nucleotide diversity (*π*), and haplotype diversity (*h*) using DnaSP 6 (Rozas et al., 2017) excluding sites with gaps. Genealogical relationships of the haplotypes were analyzed by constructing of a median-joining network in PopART (Leigh and Bryant, 2015).

### Phylogenetic Analysis

To explore the phylogenetic placement of our sampled honeybees, the Bayesian inference (BI) and maximum likelihood (ML) trees of *A*. *mellifera* subspecies were reconstructed. Mitogenome data of 21 *A*. *mellifera* subspecies and a closely related species (*A*. *cerana*) in GenBank (Supplementary Table S2) were combined with our newly sequenced mitogenomes. All genes were aligned using MUSCLE (Edgar, 2004) except the protein-coding genes, which were converted into a codon alignment by PAL2NAL (Suyama et al., 2006) based on the corresponding protein sequence alignment from MUSCLE (Edgar, 2004). The 37 gene sequences were each filtered by the Gblocks server (Castresana, 2000) to remove poorly aligned positions. The resultant sequences were concatenated into a single dataset for the BI and ML tree reconstruction. Based on 63 pre-defined partitions for each gene and codon positions, best-fit partition schemes and substitution models were determined by Bayesian Information Criterion in PartitionFinder 2 (Lanfear et al., 2017) with unlinked branch lengths and greedy search algorithms. The HKY + I model and a single partition for the whole dataset were selected and used for the BI tree reconstruction. The BI tree was built in MrBayes v3.2 (Ronquist et al., 2012) with the specified settings (two runs, 10 million generations sampled every 1000 steps, and burn-in rate of 0.25). Convergence of the two runs were supported by the average standard deviation of split frequencies (<0.01) and the potential scale reduction factor (1.00) of each parameter. The ML tree was reconstructed in IQ-TREE 2.1.1 (Minh et al., 2020) with 1000 bootstrap replicates and the best-fit partitions and models using the “TESTMERGE” option (Kalyaanamoorthy et al., 2017). Bayesian posterior probabilities and the ML bootstrap values were used as nodal supports for the BI and ML trees, respectively.

### Analyses of Selection on mtDNA

The *ω* value, the ratio of the nonsynonymous (*d*_N_) to synonymous (*d*_S_) substitution rate, is widely used to detect selection. Negative, neutral, and positive selection are inferred with *ω* < 1, *ω* = 1, and *ω* > 1, respectively (Hughes and Nei, 1988). To test for signatures of selection on mitochondrial protein-coding genes, we first calculated *ω* values using DnaSP 6 (Rozas et al., 2017). Due to lack of synonymous substitutions between some pairwise sequences, we calculated the ratio of *d*_N_ ∕ (*d*_S_ + constant), where the constant was of the *d*_S_ for one synonymous substitution. This could avoid dividing by zero as per Mishmar et al. (2003).

We performed the branch-model test by CodeML in PAML4 (Yang, 2007) to estimate the *ω* ratios for the branch of interest. To avoid potential effects of genetically distant outgroup taxa, only the 130 newly sequenced honeybees were included. To do so, the BI tree obtained above was manually modified. Due to the rejection of reciprocal monophyly of RJBs and ITBs but the main division into the RJB- and ITB-dominant sublineages (*Section Phylogenetic Reconstruction*), we compared the three-*ω* branch model (the RJB-dominant sublineage, the ITB-dominant sublineage, and the remaining) and the M0 (one-ratio) model. The two-*ω* branch model (the RJB-dominant sublineage and the remaining) and the M0 model were also compared. Significant difference between the models was assessed by the likelihood ratio test (LRT).

The branch-site model was applied to identify positively selected sites (Yang, 2007). The analysis was performed separately on genes with fixed nonsynonymous substitutions in the RJB-dominant sublineage (the foreground). Model A modified (positive selection along foreground branches) and a null model that allows neutral evolution and negative selection were compared in LRTs. Posterior probability was calculated using the Bayes empirical Bayes (BEB) method. Sites with a significant difference in LRTs and a posterior probability >0.95 were identified to be under positive selection.

### Statistical Analysis

Quantitative data were presented as means ± standard error of mean (SEM). Significant difference was determined at *p* < 0.05 by Student’s *t*-test for two groups.

## Results

### Comparison of Royal Jelly Yield

**FIGURE 1:**
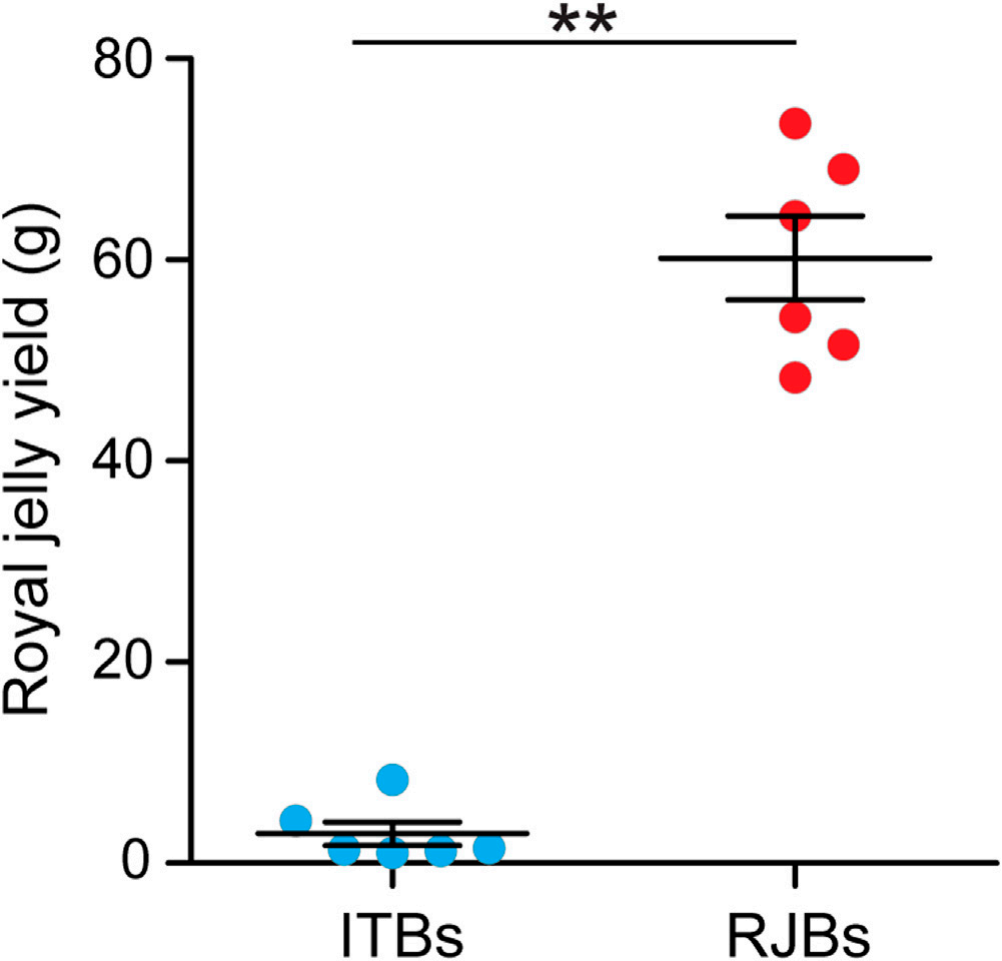
Comparison of royal jelly yield between RJBs and ITBs. Royal jelly was collected at 72 h after grafting young worker larvae into queen cells. The data are expressed as mean ± SEM (*n* = 6).

The royal jelly yield is 60.189 ± 4.186 g and 2.943 ± 1.178 g for RJBs and ITBs, respectively (Figure 1). RJBs exhibits a significantly higher capacity to produce royal jelly than do ITBs (*p* = 0.002).

### mtDNA Copy Number

**FIGURE 2:**
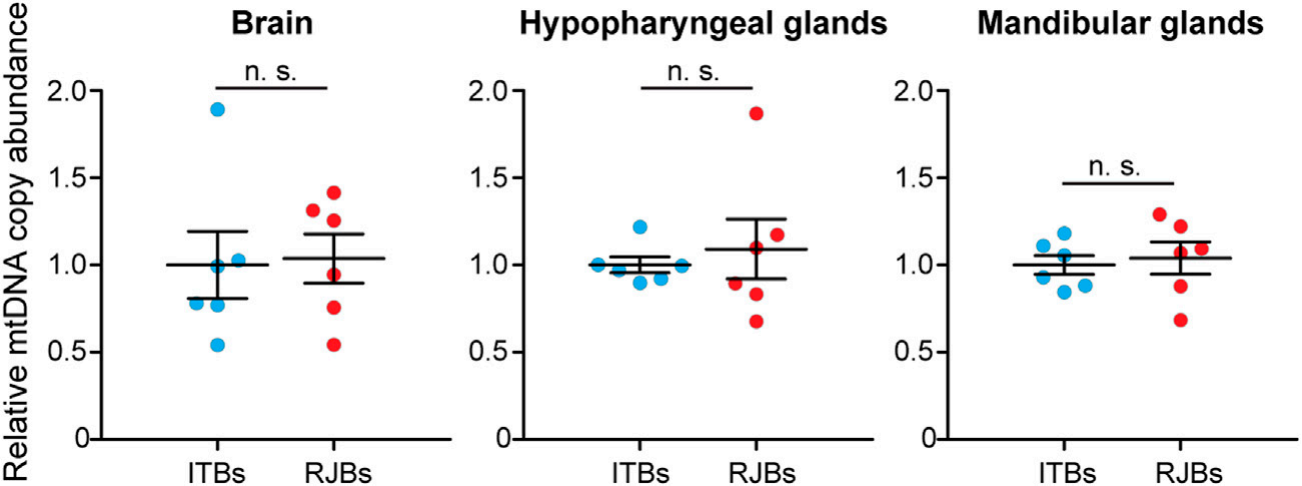
Relative mtDNA copy number between RJBs and ITBs. mtDNA copy number was compared in the brain, hypopharyngeal glands, and mandibular glands. The mtDNA copy abundance in RJBs was normalized to that (mean = 1.0) in ITBs (mean ± SEM, *n* = 6 for each strain). No significant difference was found.

To quantify the mtDNA copy abundance, qRT-PCR of a mitochondrial-encoded gene (16S RNA) and nuclear GAPDH was conducted. The resultant qRT-PCR product sequences are identical to those of 16S RNA (NC001566) and GAPDH (NC037647) in GenBank, validating accurate amplification of targeted genes. The mtDNA copy number in the brain (*p* = 0.879), hypopharyngeal glands (*p* = 0.818), and mandibular glands (*p* = 0.720) is not significantly different between RJBs and ITBs (Figure 2).

### Mitogenome Features and Genetic Diversity

**FIGURE 3:**
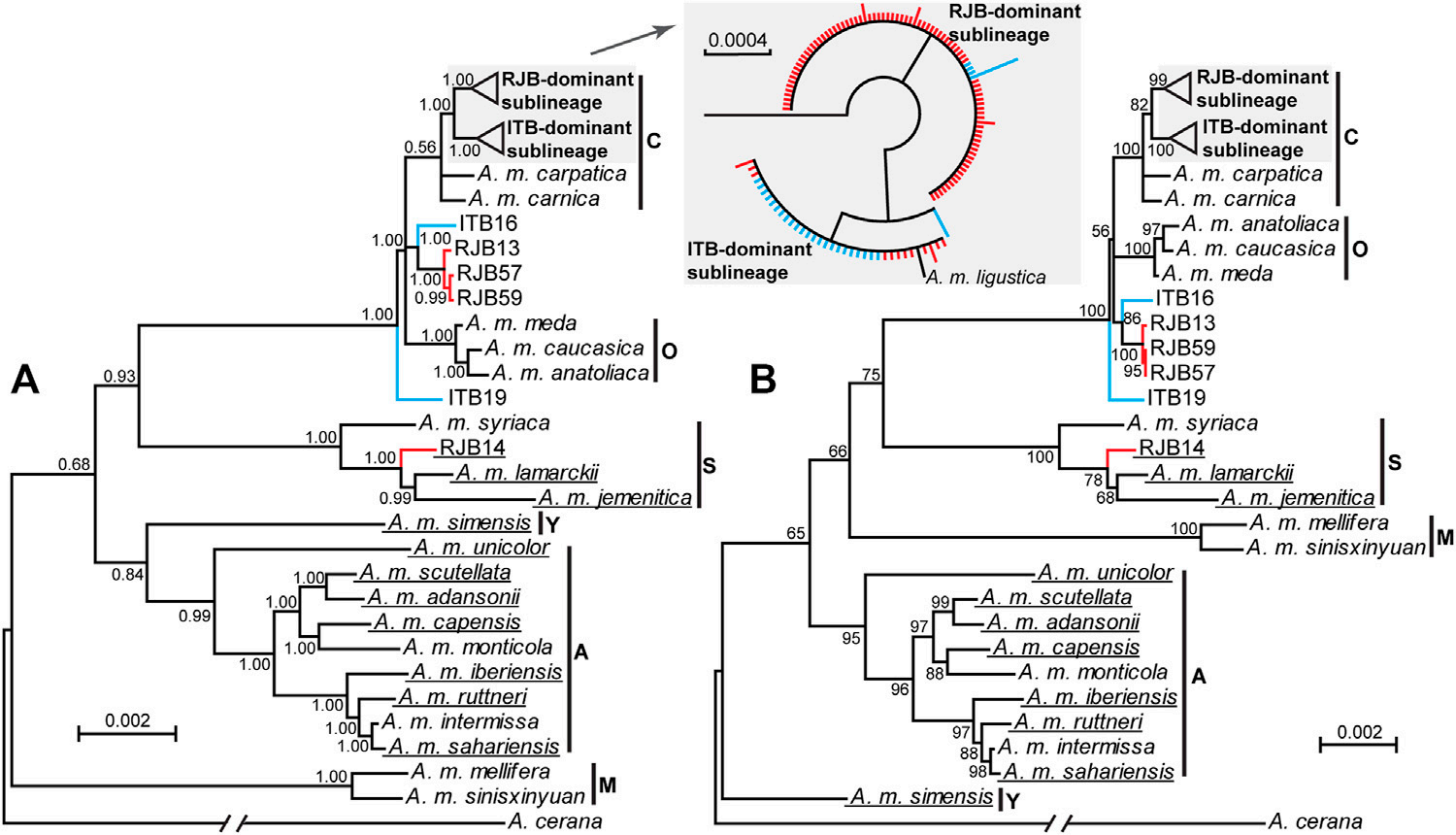
The phylogenetic trees of *A*. *mellifera* subspecies. Posterior probabilities for the Bayesian inference tree **(A)** and bootstrap values for the maximum likelihood tree **(B)** are shown at nodes. The branch of the outgroup *A*. *cerana* is truncated. Subspecies with long insert in the intergenic spacer between *cox2* and *trnL2* are underlined. The previously designated six lineages (A, C, M, O, S, and Y) for *A*. *mellifera* are indicated. The clades for the RJB- and ITB-dominant sublineages are shaded. The clustering pattern of the two sublineages is similar in the two trees and is shown in the inset (red color for RJBs and blue for ITBs) for the Bayesian inference tree.

We sequenced and assembled mitogenomes with all 37 mitochondrial genes from 100 RJBs and 30 ITBs. We failed to assemble the control region due to the extremely high A + T content, which is 96.00% in the reference mitogenome of *A*. *m*. *ligustica* (GenBank no. NC001566). These mitogenomes excluding the control region range in size from 15,489 bp to 15,647 bp with a high A + T content (84.25%–84.36%). The 37 genes are arranged in the same order as that of the reference mitogenome of *A*. *m*. *ligustica*. Strikingly, a 66-bp insert in the intergenic spacer between *cox2* and *trnL2* is exclusively present in RJB14 among all our newly sequenced mitogenomes. In a broader context of *Apis mellifera*, this insert is observed in 10 subspecies currently available in GenBank (Figure 3).

Overall, the 130 *A*. *m*. *ligustica* mitogenome sequences are highly conserved. Analysis of the 14,656-bp coding sequences (i.e., concatenated sequences of 37 genes excluding alignment gaps) yields 14 haplotypes and 224 polymorphic sites (182 singleton variable sites and 42 parsimony informative sites). The overall nucleotide (*π*) and haplotype (*h*) diversity are 0.00069 ± 0.00019 and 0.521 ± 0.041, respectively. The diversity indices are similar between ITBs (*h* = 0.411 ± 0.110, *π* = 0.00056 ± 0.00020) and RJBs (*h* = 0.336 ± 0.059, *π* = 0.00055 ± 0.00024) with no fixed nucleotide substitution between them.

### Haplotype Network Analysis

**FIGURE 4:**
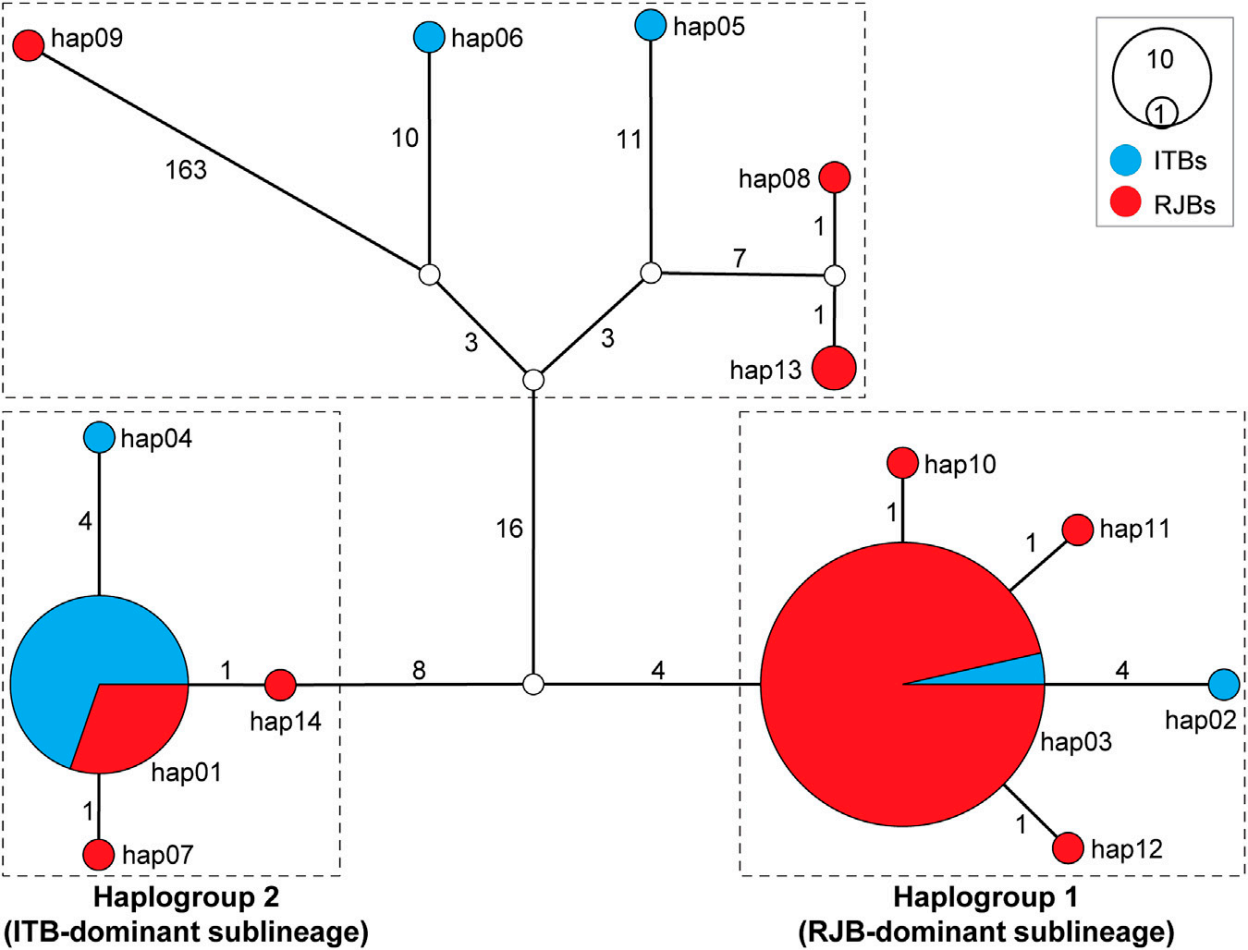
Haplotype network of RJBs and ITBs. The network was inferred from concatenated data of the 37 mitochondrial genes. Circle size is proportional to haplotype frequency. Empty circles represent hypothetical intermediate haplotypes. Mutation steps are shown next to branches. Two major haplogroups were identified with haplogroup 1 and 2 corresponding to RJB- and ITB-dominant sublineages (Figure 3), respectively.

To visualize genealogical relationships of the 14 haplotypes, a haplotype network was constructed (Figure 4). The resulting network identifies two main haplogroups (1 and 2), which are delineated by a total of 12 substitutions. Both haplogroups occur in RJBs and ITBs, but haplogroup 1 predominates in RJBs (84/100) and haplogroup 2 predominates in ITBs (24/30). Furthermore, each haplogroup is dominated by a core haplotype. Specifically, hap03 and hap01, which are separated by 1−4 substitutions from related haplotypes, serve as the core haplotype of haplogroup 1 and 2, respectively. Hap03 represents the most frequent haplotype and is shared by 81 RJB and 3 ITB bees, whereas hap01 is shared by 10 RJB and 23 ITB bees. Moreover, the haplotype network identifies five genetically discrete haplotypes, which have more than 30 substitutions relative to the two haplogroups. Notably, hap09 (RJB14) is found to delineate from other haplotypes with more than 163 substitutions.

### Phylogenetic Reconstruction

The BI and ML trees of all currently available *A*. *mellifera* subspecies were reconstructed to determine phylogenetic position of the RJBs and ITBs. If not considering the newly sequenced RJBs and ITBs, the lineages of *A*. *mellifera* are each recovered as monophyletic (Figure 3). Among them, the S lineage (Tihelka et al., 2020) is comprised of *A*. *m*. *jemenitica*, *A*. *m*. *lamarckii*, and *A*. *m*. *syriaca*. In both trees, the phylogenetic position of C, O, and S lineages is concordant, whereas the placement of A, M, and Y lineages is unresolved. The phylogenetic position of RJBs and ITBs is congruent between the two trees but neither strain is recovered as a monophyletic group. Rather, both RJBs and ITBs fall into two highly supported major clades, the RJB-dominant sublineage (84 RJBs and 4 ITBs) and the ITB-dominant sublineage (24 ITBs and 12 RJBs as well as the reference mitogenome of *A*. *m*. *ligustica*), corresponding to the two haplogroups in the haplotype network (Figure 4). The two sublineages have a close relationship with *A*. *m*. *carpatica* and *A*. *m*. *carnica*. Four RJBs (RJB13, RJB14, RJB57, and RJB59) and two ITBs (ITB16 and ITB19), belonging to the five genetically discrete haplotypes in the haplotype network (Figure 4), exhibits higher genetic differentiation from the two major sublineages. Among them, RJB14 is strongly supported to be sister to *A*. *m*. *lamarckii* and *A*. *m*. *jemenitica* in the S lineage, while ITB19 is sister to (lineage C + lineage O + the four other bees). Collectively, our newly sequenced RJBs and ITBs are comprised of two highly differentiated sublineages (the RJB- and ITB-dominant sublineages) and six divergent individuals.

### Selection Test

The 13 protein-coding genes of the RJB- and ITB-dominant sublineage exhibit low levels of sequence variation. A total of 12 synonymous and five nonsynonymous substitutions are observed with only three nonsynonymous substitutions (codon 14 and 62 of *nad2* and codon 288 of *nad4*) fixed in the RJB-dominant sublineage. The lack of sequence variation results in rather low *ω* values (0.0041 and 0.0066 for the RJB- and ITB-dominant sublineage, respectively) for the concatenated data of the 13 genes.

To test for selective pressures acting on mitochondrial protein-coding genes in the RJB-dominant sublineage, we performed the branch-model test by CodeML in PAML4 (Yang, 2007). In the null M0 (one-ratio) model, the uniform *ω* across the phylogenetic tree is inferred to be 0.047. The three-*ω* model (*ω* = 0.146 for the RJB-dominant sublineage, *ω* = 0.039 for the ITB-dominant sublineage, and *ω* = 0.046 for others) do not differ significantly from the M0 model (*p* = 0.727 in the LRT). Likewise, the two-*ω* model test yields low *ω v*alues (0.147 for the RJB-dominant sublineage and 0.046 for other taxa) and is not preferred over the M0 model (*p* = 0.433). Furthermore, the branch-site model was applied to identify positively selected sites for the RJB-dominant sublineage. This test was performed separately on *nad2* and *nad4* with fixed nonsynonymous substitutions in the RJB-dominant sublineage. However, these sites are not supported to be positively selected (posterior probability <0.70, *p* > 0.05).

## Discussion

To investigate evolutionary forces acting on mitogenomes of RJBs that have a higher energy demand due to the >10-fold higher yield of royal jelly (Figure 1), here nearly complete mitogenomes from 100 RJBs and 30 ITBs were sequenced and assembled. The network and phylogenetic analysis reject reciprocal monophyly of RJBs and ITBs but support the division into the RJB- and ITB-dominant sublineages as well as six divergent individuals. The RJB-dominant sublineage is highly enriched in RJBs (84/100) relative to ITBs (4/30). The selection test does not show evidence for positive selection on mitochondrial genes in the RJB-dominant sublineage. Moreover, mtDNA copy number is not significantly different between RJBs and ITBs.

### Phylogenetic Relationships of Major Lineages in *A. mellifera*

Due to the large and diverse distribution range of *A*. *mellifera* in Africa, Asia, and Europe, up to 33 morphologically and geographically distinct subspecies have been designated so far (Ilyasov et al., 2020). These subspecies were traditionally divided into four major lineages (A, C, M, and O) in earlier morphometric and genetic studies. Later, a fifth lineage (Y) from Ethiopia (Franck et al., 2001) and a possible sixth lineage from Syria (Alburaki et al., 2011; Alburaki et al., 2013), which was referred to as lineage S (Tihelka et al., 2020), were proposed. However, their phylogenetic relationships have long been controversial. Our phylogenetic analysis on the basis of mitogenome data supports the division of *A*. *mellifera* into the six lineages. Notably, we confirm the validity of the recently proposed S lineage (Alburaki et al., 2011; Alburaki et al., 2013), which encompasses *A*. *m*. *jemenitica*, *A*. *m*. *lamarckii*, and *A*. *m*. *syriaca*, consistent with a recent mitogenome study (Boardman et al., 2020). However, phylogenetic relationships of these lineages and, in particular, A, M, and Y still remain unresolved as is evidenced by low support values and inconsistent placements in the two trees in our study. Similar phylogenetic inconsistency has been reported in previous studies (Tihelka et al., 2020) and could be attributed to limited gene and subspecies sampling. It is expected that increased subspecies sampling, especially in Middle East and northeastern Africa with contact zones of multiple subspecies, could shed light on the phylogenetic relationships.

### Phylogenetic Placements of RJBs

Phylogenetic analysis of available *A*. *mellifera* subspecies could provide insights into phylogenetic position of the RJBs. We found that most RJBs (96/100) and ITBs (28/30) sequenced in our study cluster with the reference mitogenome of *A*. *m*. *ligustica* (NC001566) from GenBank. This observation provides ample evidence for the commonly held view that RJBs were derived from ITBs (Altaye et al., 2019; Cao et al., 2016). Moreover, the 124 bees are closely related to *A*. *m*. *carpatica* and *A*. *m*. *carnica*, consistent with previous studies (Syromyatnikov et al., 2018; Tihelka et al., 2020). In addition, we identified four divergent individuals in RJBs and two in ITBs. Among them, one RJB (RJB14) is strongly supported to belong to the lineage S, one ITB (ITB19) is sister to the clade including the lineage C and O, and the four others (ITB16, RJB13, RJB57, and RJB59) are closely related to the lineage C and O. Notably, the distinctiveness of RJB14 is also supported by the presence of a 66-bp insert between *cox2* and *trnL2*, which is also present in *A*. *m*. *lamarckii* and *A*. *m*. *jemenitica* in the S lineage (Figure 3). These findings could be used to trace the maternal origin of the divergent individuals. The presence of the two divergent ITB individuals could be due to genetic introgression from other breeds into *A*. *m*. *ligustica* populations in Italy (Minozzi et al., 2021). We propose that RJB14 maternally originated in recent years from the S lineage reared in China, while the three RJBs (RJB13, RJB57, and RJB59) were derived from the undescribed lineage containing ITB16 in Italy. An increased sampling of ITBs and other subspecies will contribute to pinpointing the maternal origin of these divergent individuals.

### Evolutionary Forces Shaping RJB Mitogenome Variation

We propose two main forces shaping mitogenome evolution during the selective breeding of RJBs. The first is purifying selection, which is strongly supported by the low *ω* value of 0.0041 [*ω* < 1 indicates purifying selection (Hughes and Nei, 1988)] for the RJB-dominant sublineage in our study. Such strong purifying selection acting on mitochondrial genes is expected due to their functional constraints in energy metabolism and has been reported in animals with increased energy demands, such as rapidly flying birds (Shen et al., 2009) and migratory fish (Sun et al., 2011). It explains the very high levels of sequence conservation in the 13 protein-coding genes with only three nonsynonymous substitutions fixed in the RJB-dominant sublineage. However, our further analysis reveals that these nonsynonymous substitutions do not exhibit signatures of positive selection. Rather, the overrepresentation of the RJB-dominant sublineage in RJBs (84/100) relative to ITBs (4/30) conforms to drift, a common evolutionary process in breeding with small founding populations (Wright et al., 2021). Therefore, we propose that genetic drift serves as a second force accounting for RJB mitogenome evolution. It could be inferred that, before the selective breeding for high royal jelly production in China, the RJB-dominant sublineage had already existed but with a low frequency in source populations in Italy. In the introduced populations to China, the RJB-dominant sublineage was probably present with an increased frequency by chance. When the breeding began, the queens of colonies screened for improved royal jelly production happened to carry mitogenomes belonging to the RJB-dominant sublineage. From this limited number of queens, further selective breeding with their daughter queens and drones continued year after year. Such genetic drift including founder effects finally leads to the 6-fold increase in the observed frequency of the RJB-dominant sublineage in RJBs relative to ITBs in our study.

### mtDNA Copy Number Comparison

An increasing body of evidence supports the alteration of mtDNA copy number in response to energy requirements and physiological conditions (Castellani et al., 2020). The mtDNA content could thus reflect the energy demand of a cell. For RJBs with increased energy metabolism, however, we did not find any significant difference in the mtDNA copy number of the brain, hypopharyngeal glands, or mandibular glands relative to ITBs (Figure 2). The finding rules out the association between mtDNA copy number and the augmented energy supply in RJBs. Similar findings have been reported from other studies. For example, queen larvae accomplish higher metabolic activity relative to worker larvae by significantly increasing mtDNA expression level but not copy number (Corona et al., 1999). It is likely that mitochondrial gene expression is upregulated at the transcriptional and/or translational level, thereby ensuring sufficient energy provision in RJBs.

## Data Availability

The newly sequenced mitogenomes presented in the study are publicly available. This data can be found in the GenBank database using accession numbers OM203219—OM203348.

## Supplementary Material

Supplementary Table S1 Sample information for the 100 RJBs and 30 ITBs.

Supplementary Table S2 Currently available mitogenomes from GenBank for comparative analysis.
